# Multiple Saccular Aneurysms of the Posterior Cerebral Artery With Mass Effect: A Case Report

**DOI:** 10.7759/cureus.111471

**Published:** 2026-06-25

**Authors:** Daisy Gómez, Michelle Idrovo, Erika Malla

**Affiliations:** 1 School of Medicine, Universidad del Azuay, Cuenca, ECU; 2 Private Medical Practice, BioMed, Cuenca, ECU

**Keywords:** brain edema, case reports, intracranial aneurysm, magnetic resonance angiography, posterior cerebral artery, transient ischemic attack

## Abstract

Intracranial aneurysms are arterial dilatations most commonly located in the circle of Willis, characterized by a focal outpouching of the arterial wall. Posterior cerebral artery (PCA) aneurysms are very uncommon and, in very rare cases, may present as multiple lesions. They are particularly associated with mass effect due to their proximity to critical midbrain and thalamic structures. Despite their low frequency, they are clinically relevant due to their higher risk of rupture and potential for significant mass effect. We report the case of a 51-year-old postmenopausal female who presented with a 72-hour history of right upper limb monoparesis, progressing to right hemihypoesthesia, hemiparesthesia, ipsilateral facial involvement, gait disturbance, and transient conduction aphasia. Magnetic resonance angiography revealed two saccular aneurysms in the left PCA: one partially thrombosed aneurysm in the P2 segment measuring 14.8 × 10 mm (neck 6.4 mm) and another measuring 10.5 × 9.9 mm (neck 4.2 mm), both associated with significant mass effect and vasogenic edema involving the mesencephalon, cerebral peduncle, and ipsilateral ambient cistern. Due to the patient’s autonomous decision and socioeconomic factors, conservative outpatient management was chosen following individualized assessment. Although microsurgical and endovascular options are generally preferred for PCA aneurysms considering their morphology, location, and institutional expertise, the final therapeutic decision was made through shared decision-making with the patient. The clinical presentation was consistent with a progressive and transient posterior circulation syndrome, secondary to a transient ischemic attack and vascular mass effect. Aneurysms in the posterior circulation can rarely affect language functions, as observed in this case with conduction aphasia. This case highlights the importance of early recognition of atypical presentations through appropriate clinical assessment and advanced imaging, allowing accurate diagnosis and timely therapeutic decision-making.

## Introduction

Aneurysms are pathological and permanent dilatations of the arterial wall [1]. Those located at the bifurcations of the circle of Willis, the primary arterial network at the base of the brain supplying blood to most of the cerebral hemispheres, are called intracranial or cerebral aneurysms, which have a prevalence of 3-5% [2,3]. Aneurysms are classified according to their morphology and can be fusiform, dissecting, or saccular aneurysms; the latter being the most frequent and also called berry-like aneurysms [1]. The majority of intracranial aneurysms are located in the anterior circulation, and posterior cerebral artery (PCA) aneurysms are infrequent, accounting for only 1-2% of all intracranial aneurysms [2]. The PCA supplies critical structures involved in vision, sensory processing, and midbrain function [4]. Due to their anatomical location adjacent to the midbrain, thalamus, and cerebral peduncles, this kind of aneurysm is particularly associated with mass effect when they enlarge [5]. 

PCA aneurysms are clinically challenging because they can mimic other neurological syndromes through two main mechanisms: direct mass effect on adjacent structures or transient ischemia due to thromboembolism or hemodynamic compromise [6,7]. Despite their low frequency, they are clinically relevant due to their ability to reach large sizes and their higher risk of rupture [8]. Most of these aneurysms are usually solitary; however, in 10-30% of cases, they present in a multiple form [9]. Recent studies have shown that both microsurgical and endovascular approaches can achieve favorable outcomes, although treatment must be highly individualized based on aneurysm morphology, location, and patient characteristics [10,11].

Consequently, it is important to document uncommon clinical cases, such as PCA aneurysms, due to their clinical variability. For this reason, we present the case of a 51-year-old woman diagnosed with multiple PCA aneurysms, associated with an uncommon clinical presentation, highlighting the importance of early detection to prevent potentially devastating neurological complications.

## Case presentation

A 51-year-old right-handed female patient, a housewife of middle-low socioeconomic status and a Jehovah’s Witness, presented with a 72-hour history of paresthesia in the right upper extremity, extending from the fingers to the shoulder. After 48 hours, symptoms progressed with radiation to the ipsilateral thoracoabdominal region and right lower extremity. Within the following 24 hours, she developed right upper limb monoparesis. Eight hours prior to admission, she experienced transient conduction aphasia. She denied other associated symptoms, including headache, vomiting, visual changes, or respiratory distress. Her past medical history was significant for hypercholesterolemia, bilateral patellar chondromalacia, and hypermetropia. She denied regular medication use. Gynecological and obstetric history included three pregnancies, one full-term delivery, and two spontaneous abortions of unknown cause. Menopause occurred at the age of 45 without pharmacological treatment. She denied alcohol, tobacco, or illicit drug use. Family history was notable for Parkinson's disease in her mother at age 75 and liver cancer-related death in her father.

On physical examination, the patient was hemodynamically stable: blood pressure 128/80 mmHg, heart rate 76 beats per minute, respiratory rate 18 breaths per minute, temperature 36.7°C, and oxygen saturation 95% on room air (FiO₂ 21%). The mildly reduced oxygen saturation was considered incidental and likely related to the patient’s residence at an altitude of 2,560 meters above sea level, as she had no respiratory symptoms or signs of hypoxia.

Neurological examination revealed an alert patient, oriented to time, place, and person, with preserved higher mental functions. Cranial nerves were intact, meningeal signs were negative, deep tendon reflexes were normal, and Babinski sign was negative. At the time of evaluation, speech showed no motor or expressive impairment, consistent with the transient nature of the previously reported conduction aphasia. Sensory examination demonstrated mild-to-moderate hypoesthesia (4/5), and muscle strength was 4/5 in the right hemibody. Gait was slightly deviated to the left. The National Institutes of Health Stroke Scale (NIHSS) score was 3, indicating a mild neurological deficit.

Diagnostic assessment

In the context of a central neurological syndrome, initial laboratory studies were performed and were within normal limits (Table 1). Given the suspicion of a subacute ischemic event due to the focal neurological deficit, a non-contrast brain computed tomography (CT) scan was obtained, revealing findings suggestive of an aneurysmal versus tumoral process (Figure 1).

**Table 1:** Laboratory findings at admission WBC: white blood cells; RBC: red blood cells; Hb: hemoglobin; Hct: hematocrit; ALT: alanine aminotransferase; AST: aspartate aminotransferase; TSH: thyroid-stimulating hormone; HDL: high-density lipoprotein; LDL: low-density lipoprotein

	Parameters	Results	Reference range
Blood Count	WBC	4.75 mm^3^	4.0 – 10.0
	Neutrophils	56.7%	50 – 70
	Lymphocytes	35.5%	20 – 40
	Eosinophils	2.6%	0.5 – 5.0
	Monocytes	5.2%	3 – 12
	RBC	4.63 mm^3^	3.50 – 5.00
	Hb	15 g/dl	11 – 15
	Hct	43.3 %	37 – 47
	Platelets	291 mm^3^	150 – 450
Blood Chemistry	Glucose	85.5 mg/dl	60 – 110
	Urea	17.6 mg/dl	15 – 45
	Creatinine	0.85 mg/dl	0.6 – 1.1
	Cholesterol	206.69 mg/dl	<200
	Triglycerides	229.61 mg/dl	35 – 135
	LDL Cholesterol	112.91 mg/dl	<150
	HDL Cholesterol	47.86 mg/dl	45 – 60
	ALT	28.2 U/L	<32
	AST	25.03 U/L	<31
Hormonal Studies	TSH	2.45 mIU/L	0.3 – 4.2
	T3	3.85 pg/ml	2.0 – 4.4
	T4	7.95 ng/dl	5.12 – 14.06

**Figure 1:**
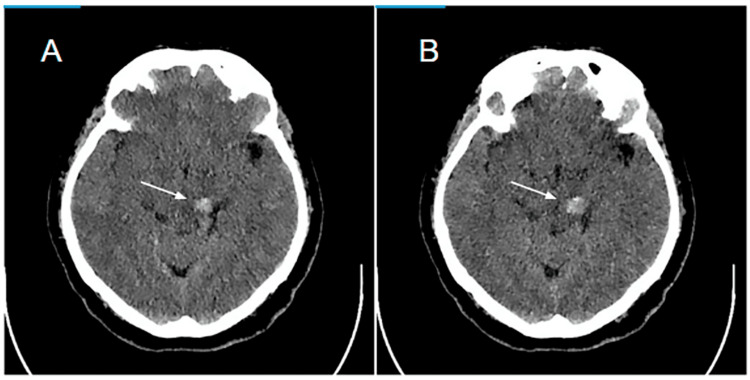
Non-contrast brain computed tomography (CT) suggestive of aneurysm. (A, B) Axial CT images showing a nodular hyperdense lesion in the left cerebral peduncle measuring 1.5 × 0.9 × 1.1 cm (arrow), as well as an additional hyperdense lesion extending superiorly toward the left quadrigeminal cistern, measuring 0.9 × 0.88 cm, suggestive of aneurysms.

The patient was subsequently referred to neurosurgery. Magnetic resonance angiography (MRA) was performed, which is a non-invasive modality preferred for initial vascular and soft tissue evaluation. This study confirmed the diagnosis of two saccular aneurysms in the left PCA, associated with mass effect and vasogenic edema involving the mesencephalon, left cerebral peduncle, and ambient cistern (Figure 2). Although digital subtraction angiography (DSA) remains the gold standard for detailed assessment of branch anatomy, neck configuration, and treatment planning in complex aneurysms, it was not performed initially due to the patient’s religious beliefs as a Jehovah’s Witness (refusal of invasive procedures with potential bleeding risk), combined with socioeconomic limitations and the fact that MRA provided sufficient diagnostic information for initial management and outpatient follow-up.

**Figure 2:**
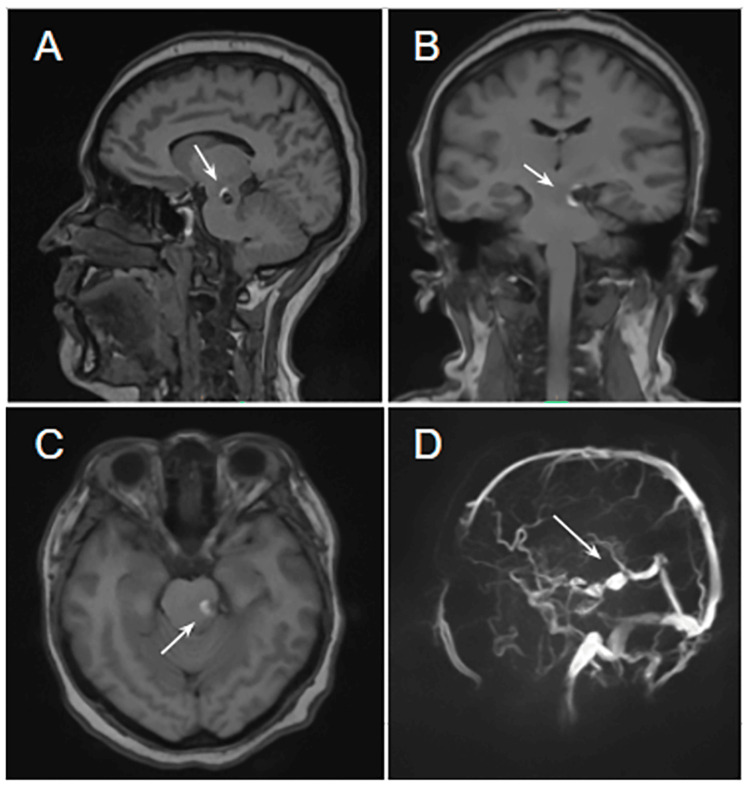
Contrast-enhanced magnetic resonance angiography (MRA) of the brain showing saccular aneurysms of the left posterior cerebral artery. (A–C) Sagittal, coronal, and axial views demonstrating a saccular aneurysm in the P2 segment measuring 14.8 × 10 mm with a 6.4 mm neck, showing peripheral partial thrombosis and associated mass effect on the left mesencephalon with marked vasogenic edema (white arrows). (D) Three-dimensional angiographic reconstruction showing an additional saccular aneurysm measuring 10.5 × 9.9 mm with a 4.2 mm neck, located in the left ambient cistern, without evidence of thrombosis (arrowhead).

The presence of neurological deficits suggested recent aneurysmal expansion. Risk assessment was performed using the ELAPSS (Earlier subarachnoid hemorrhage, aneurysm Location, Age, Population, aneurysm Size and Shape) [12], UIATS (Unruptured Intracranial Aneurysm Treatment Score) [13], PHASES [14], and ABCD2 [15] scores. Although these scales were originally developed and validated primarily for incidental or asymptomatic unruptured intracranial aneurysms, they were applied in this case due to the absence of more specific tools for symptomatic aneurysms with mass effect and thrombosis. These scores helped estimate the risk of aneurysm growth and rupture, as well as the potential benefit of intervention versus conservative management. We acknowledge their limitations in this specific clinical context, particularly because the patient was symptomatic with mass effect and partial thrombosis, conditions not fully accounted for in the original design of these scales. Nevertheless, they provided a useful structured framework for decision-making when combined with clinical judgment and patient preferences (Table 2).

**Table 2:** Scales for risk assessment and management of intracranial aneurysms Note: These scoring systems were primarily designed for incidental unruptured intracranial aneurysms. Their application in symptomatic cases with mass effect and partial thrombosis should be interpreted with caution. ELAPSS (Earlier subarachnoid hemorrhage, aneurysm Location, Age, Population, aneurysm Size and Shape): Estimates risk of aneurysm growth; UIATS (Unruptured Intracranial Aneurysm Treatment Score): Helps in treatment decision-making (surgery vs conservative); PHASES: Predicts five-year rupture risk; ABCD2: Assesses short-term stroke risk after transient ischemic attack.

Scale	Aneurysm 1	Aneurysm 2	Interpretation
ELAPSS [12]	31 points	31 points	Growth risk: 42% at 3 years, 60.8% at 5 years
UIATS [13]	14 vs 12	23 vs 8	Favoring surgical management
PHASES [14]	10 points	10 points	5.3% rupture risk at 5 years (intermediate risk)
ABCD2 [15]	2 points	2 points	Estimated 90-day stroke risk: 3.1% (low risk)

Follow-up

During follow-up evaluations, performed on a weekly basis, the patient reported intermittent episodes of conduction aphasia during daily activities. These episodes were attributed to transient ischemic attacks (TIAs), secondary to microemboli from the partially thrombosed aneurysm and the hemodynamic steal phenomenon. Motor, sensory, and gait disturbances previously observed in the right hemibody had resolved. Residual findings included mild hypoesthesia in the right mandibular region (V3 branch of the trigeminal nerve) and in the medial arch and metatarsal area of the right foot, consistent with mass effect on the sensory pathways at the mesencephalic level. The chronological evolution of symptoms is shown in Figure 3.

**Figure 3:**
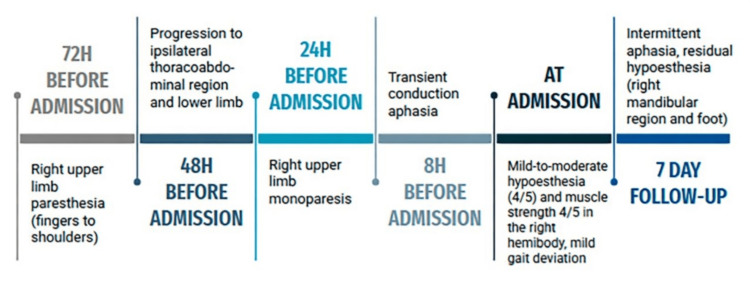
Chronology of the patient's clinical evolution H: hours

Treatment

Although the standard of care for symptomatic large PCA aneurysms with mass effect and partial thrombosis is interventional treatment (either endovascular or microsurgical), the patient declined these options. Due to her religious beliefs as a Jehovah’s Witness, which include refusal of blood transfusions, and her socioeconomic situation, she opted for expectant conservative management. This decision was made after extensive shared decision-making, clearly explaining the risks of rupture and neurological deterioration.

The patient chose to pursue homeopathic therapy, including compounded medications, probiotics, amino acids, trace elements, and unspecified intravenous therapy, against medical recommendation. This approach is not evidence-based and is not supported by current guidelines for the management of intracranial aneurysms. She was advised on the importance of strict blood pressure control and was prescribed antiplatelet therapy (aspirin 100 mg daily) due to the presence of partial thrombosis in one of the aneurysms, while awaiting follow-up neuroimaging.

## Discussion

In the presence of a central neurological syndrome characterized by motor, sensory, and language involvement, five main differential diagnoses were considered: degenerative, infectious, traumatic, tumoral, and vascular etiologies. The first three were initially ruled out based on clinical evaluation and initial laboratory findings. Although there was a family history of Parkinson’s disease, the patient did not exhibit systemic motor alterations, prodromal features, cognitive impairment, or a history of gradual or progressive neurological decline [16]. There was no evidence of fever, meningeal signs, altered mental status, headache, or laboratory findings suggestive of infection [17]. Additionally, the patient denied any history of head trauma, and no clinical signs supported this diagnosis.

Based on this analysis, a tumoral (primary vs. secondary) and/or vascular origin (arteriovenous malformations, stroke, aneurysms) was considered more likely, given the presence of a subacute focal neurological deficit, family history of cancer, associated risk factors, and unremarkable laboratory results [18].

Clinical assessment initially guided the request for a non-contrast brain CT scan, which supported the suspicion of a mass lesion versus a vascular abnormality. Subsequently, MRA was performed, confirming the diagnosis of two unruptured saccular aneurysms of the PCA. The main risk factors identified in this patient included age over 50 years, female sex, and possible estrogen deficiency [19].

From a clinical standpoint, the patient presented with a transient and progressive left posterior circulation syndrome, predominantly involving the PCA territory. Initially, she developed moderate right upper limb monoparesis, which is an uncommon manifestation of PCA involvement [20]. Within hours, this progressed to mild right hemihypoesthesia and hemiparesthesia due to impaired perfusion of thalamic perforating branches. Mild facial hypoesthesia on the right side is explained by a mass effect on the ipsilateral trigeminal nucleus at the mesencephalic level [21]. Subsequently, intermittent conduction aphasia developed due to involvement of the ipsilateral parieto-occipital region in the dominant (left) hemisphere, given that the patient is right-handed [22]. This finding is particularly noteworthy, as conduction aphasia classically results from lesions affecting the arcuate fasciculus in the dominant hemisphere. During the course of the disease, the patient also presented a transient deviation of gait toward the affected side, another uncommon feature in PCA pathology [20]. It is important to note that no language impairment was observed during physical examination, and most symptoms resolved during follow-up.

Saccular aneurysms are characterized by structural vascular damage, including thinning of the vessel wall, partial or complete absence of the tunica media, and fragmentation or absence of the internal elastic lamina. These changes lead to turbulent blood flow, favoring thrombosis, embolism, and vasospasm, which contribute to the development of TIAs. Additionally, progressive endothelial degeneration and inflammation can disrupt the blood-brain barrier, resulting in vasogenic edema and progressive enlargement of the lesion, leading to mass effect on adjacent structures [23].

Based on the pathophysiology of the disease and the patient’s clinical course characterized by a subacute, progressive, and intermittent presentation with partial symptom resolution, the clinical picture is explained by an initial TIA of both embolic origin (due to aneurysmal thrombus) and vasospastic mechanisms, followed by a mass effect secondary to both aneurysm morphology and vasogenic edema [23]. The intermittent conduction aphasia observed during follow-up is a noteworthy feature of this case. Its recurrent nature suggests ongoing embolic phenomena from the thrombosed aneurysmal sac or a local hemodynamic disturbance. Additionally, the distribution of residual hypoesthesia (right V3 trigeminal territory and right foot) correlates anatomically with the mass effect exerted on the mesencephalon and lemniscal pathways, further supporting the relationship between the imaging findings and the clinical presentation.

Regarding management, although current guidelines and risk scores (ELAPSS, UIATS, PHASES) generally favor interventional treatment (microsurgical clipping or endovascular embolization) for symptomatic large PCA aneurysms with mass effect and partial thrombosis, this patient ultimately underwent conservative management. This decision represented a significant ethical and clinical challenge. The patient’s religious beliefs, which included the refusal of blood transfusions, played a central role in her autonomous decision to decline invasive procedures due to the perceived risk of bleeding. After a thorough discussion of the risks and benefits, including the potential for aneurysm rupture and further neurological deterioration, expectant management was respected in accordance with the patient’s values and wishes.

It is important to recognize the persistent risk of recurrence and de novo aneurysm formation despite surgical intervention, which requires long-term follow-up. In cases of conservative management, imaging surveillance and patient education are essential. Follow-up should include periodic assessment using the ELAPSS score and imaging studies such as CT angiography or MRA at six months, annually for two to three years, and then every two to five years if clinical and radiological stability is maintained. Patient education should address warning signs and risk factors, including the risk of subarachnoid hemorrhage, sudden severe headache, hypertension control, avoidance of alcohol, tobacco, and illicit drugs, as well as limiting excessive physical exertion and Valsalva maneuvers [23,24]. The patient was prescribed antiplatelet therapy (aspirin 100 mg daily) to reduce the risk of thromboembolic events from the partially thrombosed aneurysm. No corticosteroids were administered for the vasogenic edema. Strict blood pressure control was emphasized. During follow-up, she continued with homeopathic therapy of her choice, against medical recommendation.

Limitations

The PHASES score has not been validated in our population; however, due to the lack of locally validated tools and considering the clinical context, its use was deemed appropriate [20,23]. Additionally, values for the UIATS score were adapted using MRA due to the unavailability of conventional angiography [14]. Another important limitation is that the patient opted for homeopathic treatment against medical recommendation, which limits the generalizability of the therapeutic approach presented in this case. Furthermore, the follow-up period remains limited, with no long-term imaging studies or extended clinical outcomes available, as the patient declined further invasive evaluations or surgical intervention in favor of conservative management.

## Conclusions

PCA aneurysms are an uncommon clinical entity and often have a non-specific presentation, which may mimic other neurological syndromes. When unruptured, they may remain asymptomatic for long periods until they reach a large size, at which point symptoms are related to the mass effect they produce. Due to their location, the clinical presentation in this case mimicked a transient posterior cerebral ischemic syndrome, which explains the intermittent nature of the symptoms. Early MRA was essential for diagnostic confirmation and as a tool for treatment planning. According to the applied risk scores and current literature, surgical management (microsurgical or endovascular) was the recommended treatment. However, the patient chose conservative homeopathic treatment. This decision poses both clinical and ethical challenges; therefore, her clinical evolution and imaging follow-up will be closely monitored, and it will be a topic for a future publication. This case highlights the importance of early recognition of atypical presentations, as well as the need to respect patient autonomy in complex bioethical decisions.

## References

[1] Wiebers DO, Piepgras DG, Meyer FB (2004). Pathogenesis, natural history, and treatment of unruptured intracranial aneurysms. Mayo Clin Proc.

[2] Schievink WI (1997). Intracranial aneurysms. N Engl J Med.

[3] Deniwar M (2022). Management of multiple and unruptured cerebral aneurysms. Egypt J Neurosurg.

[4] Hamada J, Morioka M, Yano S, Todaka T, Kai Y, Kuratsu J (2005). Clinical features of aneurysms of the posterior cerebral artery: a 15-year experience with 21 cases. Neurosurgery.

[5] Stehbens WE (1963). Aneurysms and anatomical variation of cerebral arteries. Arch Pathol.

[6] Lawton MT, Vates GE (2017). Subarachnoid hemorrhage. N Engl J Med.

[7] Jabeen S, Pendharkar HS, Prasad C, Hr A, Saini J, Gupta AK, Shukla D (2021). Dissecting aneurysms of the posterior cerebral artery - a retrospective review of imaging, angiographic characteristics, endovascular management and outcome. Interv Neuroradiol.

[8] Vlak MH, Algra A, Brandenburg R, Rinkel GJ (2011). Prevalence of unruptured intracranial aneurysms, with emphasis on sex, age, comorbidity, country, and time period: a systematic review and meta-analysis. Lancet Neurol.

[9] Essibayi MA, Oushy SH, Keser Z, Lanzino G (2022). Natural history and management of posterior cerebral artery aneurysms: a systematic review and meta-analysis of individual patient data. Neurosurg Rev.

[10] Li XY, Li CH, Wang JW, Liu JF, Li H, Gao BL (2021). Endovascular management of cerebral aneurysms of the posterior cerebral artery. Front Neurol.

[11] Friedman JA, Piepgras DG, Pichelmann MA, Hansen KK, Brown RD Jr, Wiebers DO (2001). Small cerebral aneurysms presenting with symptoms other than rupture. Neurology.

[12] Backes D, Rinkel GJ, Greving JP (2017). ELAPSS score for prediction of risk of growth of unruptured intracranial aneurysms. Neurology.

[13] Etminan N, Brown RD Jr, Beseoglu K (2015). The unruptured intracranial aneurysm treatment score: a multidisciplinary consensus. Neurology.

[14] Greving JP, Wermer MJ, Brown RD (2014). Development of the PHASES score for prediction of risk of rupture of intracranial aneurysms: a pooled analysis of six prospective cohort studies. Lancet Neurol.

[15] Johnston SC, Rothwell PM, Nguyen-Huynh MN (2007). Validation and refinement of scores to predict very early stroke risk after transient ischaemic attack. Lancet.

[16] Postuma RB, Berg D, Stern M (2015). MDS clinical diagnostic criteria for Parkinson's disease. Mov Disord.

[17] van de Beek D, de Gans J, Spanjaard L, Weisfelt M, Reitsma JB, Vermeulen M (2004). Clinical features and prognostic factors in adults with bacterial meningitis. N Engl J Med.

[18] McNeill KA (2016). Epidemiology of brain tumors. Neurol Clin.

[19] Longstreth WT, Nelson LM, Koepsell TD, van Belle G (1994). Subarachnoid hemorrhage and hormonal factors in women. A population-based case-control study. Ann Intern Med.

[20] Strbian D, Sundararajan S (2013). Young patient with aphasia, monoparesis, facial drop, facial sensory loss, diplopia, dysarthria, and vertical gaze palsy. Stroke.

[21] Kim JS, Lee JH, Lee MC (1997). Patterns of sensory dysfunction in lateral medullary infarction. Clinical-MRI correlation. Neurology.

[22] Clark DG (2024). Approach to the patient with aphasia. UpToDate.

[23] Singer RJ, Ogilvy CS, Rordorf G (2026). Unruptured intracranial aneurysms. UpToDate.

[24] Singer RJ, Ogilvy CS, Rordorf G (2025). Treatment of cerebral aneurysms. UpToDate.

